# Potential of different fluoride gels to prevent erosive tooth wear caused by gastroesophageal reflux

**DOI:** 10.1186/s12903-021-01548-6

**Published:** 2021-04-09

**Authors:** Philipp Körner, Luca Georgis, Daniel B. Wiedemeier, Thomas Attin, Florian J. Wegehaupt

**Affiliations:** 1 Clinic of Conservative and Preventive Dentistry, Center of Dental Medicine, University of Zurich, Plattenstrasse 11, 8032 Zurich, Switzerland; 2 Statistical Services, Center of Dental Medicine, University of Zurich, Zurich, Switzerland

**Keywords:** Dental erosion, Erosive tooth wear, Gastroesophageal reflux disease, Erosion protection, Fluoride gel

## Abstract

**Background:**

This in-vitro-study aimed to evaluate the potential of different fluoride gels to prevent gastroesophageal reflux induced erosive tooth wear.

**Methods:**

Surface baseline profiles of a total of 50 bovine enamel specimens [randomly assigned to five groups (G1–5)] were recorded. All specimens were positioned in a custom made artificial oral cavity and perfused with artificial saliva (0.5 ml/min). Reflux was simulated 11 times a day during 12 h by adding HCl (pH 3.0) for 30 s (flow rate 2 ml/min). During the remaining 12 h (overnight), specimens were stored in artificial saliva and brushed twice a day (morning and evening) with a toothbrush and toothpaste slurry (15 brushing strokes). While specimens in the control group (G1) did not receive any further treatment, specimens in G2–5 were coated with different fluoride gels [Elmex Gelée (G2); Paro Amin Fluor Gelée (G3); Paro Fluor Gelée Natriumfluorid (G4); Sensodyne ProSchmelz Fluorid Gelée (G5)] in the evening for 30 s. After 20 days, surface profiles were recorded again and enamel loss was determined by comparing them with the baseline profiles. The results were statistically analysed using one-way analysis of variance (ANOVA) followed by Tukey`s HSD post-hoc test.

**Results:**

The overall highest mean wear of enamel (9.88 ± 1.73 µm) was observed in the control group (G1), where no fluoride gel was applied. It was significantly higher (*p* < 0.001) compared to all other groups. G2 (5.03 ± 1.43 µm), G3 (5.47 ± 0.63 µm, *p* = 0.918) and G4 (5.14 ± 0.82 µm, *p* > 0.999) showed the overall best protection from hydrochloric acid induced erosion. Enamel wear in G5 (6.64 ± 0.86 µm) was significantly higher compared to G2 (*p* = 0.028) and G4 (*p* = 0.047).

**Conclusions:**

After 20 days of daily application, all investigated fluoride gels are able to significantly reduce gastroesophageal reflux induced loss of enamel.

## Background

Although great efforts have been made in the course of reducing the prevalence of caries [1], the management of dental hard tissues loss still is a main issue in modern dentistry. Especially tooth wear caused by erosion has increasingly been reported and come into focus [2]. The growing numbers might certainly be attributed to a rising prevalence [3], but can also be related to an enhanced awareness going along with specific examination and diagnosis [4]. In general, dental erosion (DE), as the primary etiological factor for erosive tooth wear, is defined as surface dissolution of dental hard tissues caused by chemical processes not involving bacteria [5]. There are various risk factors and many different ways for people of all ages to face oral acid exposure in their everyday life [6]. Anyway, the DE causing acids are either from extrinsic or intrinsic origin [7]. While most extrinsic acids get in contact with dental hard tissues during the consumption of acidic food, beverages or drugs, intrinsic acids attack dental hard tissues during vomiting or in the course of gastroesophageal reflux (GERD) [8]. Last mentioned is an anti-peristaltic process of the gastrointestinal tract where gastric fluid, which is mainly composed of hydrochloric acid (HCl), is regurgitated up the esophagus before finally reaching the oral cavity [9]. This leads to an oral pH drop to pH < 4 [10]. Getting in contact with dental hard tissues, the acids dissolve minerals from tooth surfaces causing a demineralized and softened surface layer which easily can be removed by mechanical forces [11]. Besides, a continuous layer-by-layer dissolution might result in a permanent loss of dental hard tissues [12]. In current literature the prevalence of DE in GERD patients is described between 10 and 42% (median 25.5%) [13].

Whenever possible, the first choice of interventions should be the treatment and elimination of causative factors in the sense of primary prevention. However, in patients suffering from DE caused by gastroesophageal reflux, it might be complicated or even impossible to completely stop the erosion-causing processes. Beside a cause-related therapy, these patients are in need of additional local treatment in order to minimize dental hard tissue loss [14, 15]. Different approaches aiming to support remineralisation of softened enamel and increase acid resistance have been discussed [16]. There is evidence that fluoride matches these requirements in the course of caries protection [17]. However, the potential of fluoride to reduce erosive tooth wear still is discussed controversially in literature [16, 18–20] although lately, there is more and more growing consensus, that fluorides are also able to protect dental hard tissues from DE [21]. These studies are mainly related to acid attacks simulating contact with nutritional acids, such as citric acid with a pH range of 2–3. In contrast, information about the effect of different fluoride gels, in respect to pH-value, amount and content of fluorides, on DE caused by hydrochloric acid in the course of gastroesophageal reflux are rare. Therefore, it was the aim of the present study to investigate the potential of different fluoride gels to reduce or protect from GERD-induced dental erosion.

## Methods

### Specimen preparation and allocation

A total of 50 enamel specimens were gained from the crowns of post-mortem extracted bovine incisors received from a local slaughterhouse and stored in tap water until use. Cylindrical enamel specimens (3 mm diameter) were prepared using a diamond trephine mill (BFW 40/E, PROXXON; Föhren, Germany) and centrally embedded in acryl resin (Paladur, Kulzer; Hanau, Germany) to enable sufficient fixation during profilometric surface scan. In an automatic grinding machine with 5 N pressure, 150 rpm and water cooling (Tegramin 30, Struers; Birmensdorf, Switzerland), specimens’ enamel surfaces were ground flat in three steps using carborundum discs (SiC Foil, Struers) with decreasing grain size (1000 grit, 10 s; 2000 grit, 20 s; 4000 grit, 40 s). Using a Knoop hardness measuring device (High Quality Hardness Tester, Buehler; Düsseldorf, Germany), microhardness of each specimen was determined by performing three indentations on the respective enamel surface (load weight 50 g, indentation time 20 s). According to their mean microhardness, the prepared specimens were subsequently stratified and allocated to five groups (G1–5), labelled, and stored in tap water. Tooth collection was carried out in accordance with relevant guidelines and regulations. Additional approval by the associated Swiss Ethics Committee was not required.

### Experimental procedure

**Fig. 1:**
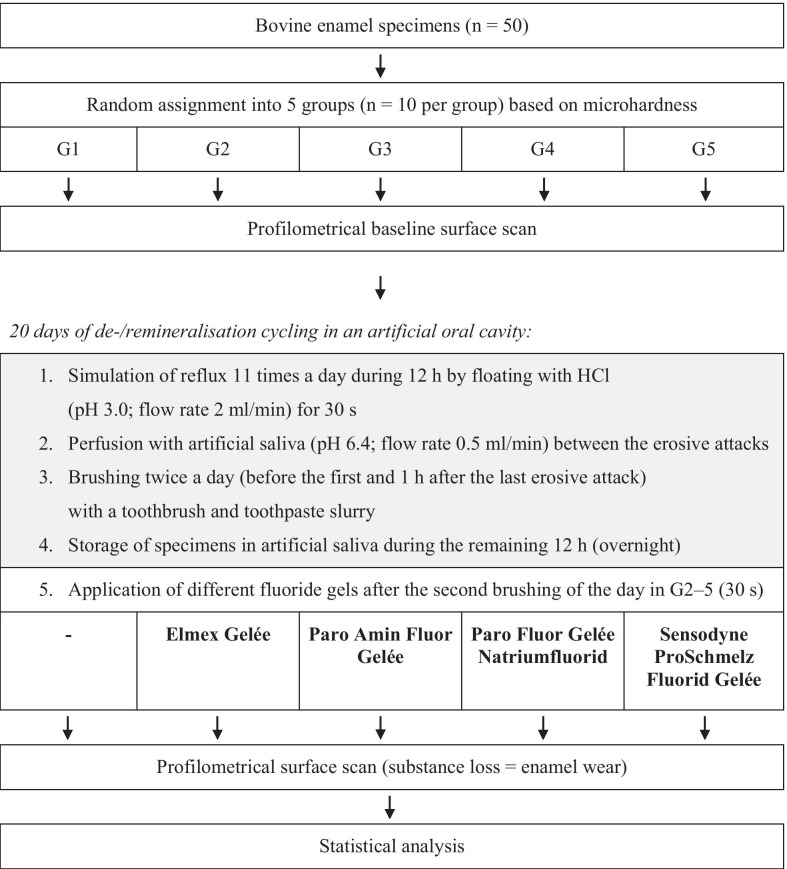
Experimental design

Initially, surface baseline profiles of all specimens were recorded (see profilometric analysis). Subsequently, the de-/remineralisation cycling was performed in a custom made artificial oral cavity which has previously been described in detail [22]. Reflux was simulated 11 times a day during 12 h by pouring a total of 1 ml of acid (HCl, pH 3.0) continuously over each enamel specimen for 30 s (flow rate 2 ml/min). Between the erosive attacks, specimens were perfused with artificial saliva (pH 6.4; flow rate 0.5 ml/min) which was formulated according to Klimek et al. [23] and renewed every day. While immediately after each attack, the flow rate was enhanced (20.0 ml/min, 3 s) to simulate increased salivary flow during acid exposure in-vivo [24] and to wash away the acid and stop the erosive process, the regular flow rate between the erosive attacks was 0.5 ml/min which corresponds with normal unstimulated salivation [25]. During the remaining 12 h (overnight), specimens were stored in artificial saliva and brushed twice a day (before the first and 1 h after the last erosive attack) with a toothbrush (Paro M43, Esro AG; Kilchberg, Switzerland) and toothpaste (Elmex Caries Protection, GABA; Therwil, Switzerland) slurry (mix of toothpaste and artificial saliva at a weight ratio of 1:2). Each time, a total of 15 brushing strokes (1 stroke/second) with a constant brushing load of 2.0 N were applied. After the second brushing of the day (evening), specimens in G2–5 were additionally coated with different fluoride gels [G2: amine/sodium fluoride, pH 4.8 (Elmex Gelée, GABA); G3: amine/sodium fluoride, pH 4.5–5.0 (Paro Amin Fluor Gelée, Esro AG); G4: sodium fluoride, pH 6.7–7.3 (Paro Fluor Gelée Natriumfluorid; Esro AG); G5: sodium fluoride, pH 5.7 (Sensodyne ProSchmelz Fluorid Gelée, GSK AG; Rotkreuz, Switzerland)] for 30 s and rinsed with water. All four fluoride gels had a F^−^-concentration of 12.500 ppm (12.5 mg per 1 g gel). Specimens in the control group (G1) did not receive any further treatment. After 20 days, surface profiles of all samples were recorded a second time and enamel loss was determined by comparing them with the baseline profiles (see profilometric analysis). The study design is illustrated in Fig. 1 and further information and details about the active ingredients of the different products (G2–5) are given in Table 1.

**Table 1:** Information and details about the active ingredients of the different products used in the study

Fluoride gels	Group	Active ingredients per 1 g gel	Total amount of F^−^-Ion per 1 g gel	pH	Manufacturer
Elmex Gelée	G2	Amine fluoride: Olaflur (30.3 mg); Dectaflur (2.9 mg) Sodium fluoride (22.1 mg)	12.5 mg	4.8	GABA; Therwil, Switzerland
Paro Amin Fluor Gelée	G3	Amine fluoride: Olaflur and Dectaflur (13.3 mg) Sodium fluoride (25.4 mg)	12.5 mg	4.5–5.0	Esro AG; Kilchberg, Switzerland
Paro Fluor Gelée Natriumfluorid	G4	Sodium fluoride (27.6 mg)	12.5 mg	6.7–7.3	Esro AG; Kilchberg, Switzerland
Sensodyne ProSchmelz Fluorid Gelée	G5	Sodium fluoride (27.6 mg)	12.5 mg	5.7	GSK AG; Rotkreuz, Switzerland

### Profilometric analysis

Before baseline profiles were recorded, two parallel reference lines with a distance of 3.4 mm were notched in the embedding acryl resin of each specimen close to the enamel margin. Additionally, profilometer and specimens were fitted with a jig to enable exact repositioning. From each specimen five baseline profiles with a distance of 250 μm between each profile were recorded using a stylus profilometer (Perthometer S2 Concept, Mahr; Göttingen, Germany) with a stylus force of < 0.7 mN and a lower measuring limit of < 130 nm profile difference [26]. The reference areas were covered with tape (Tesa, Beiersdorf; Hamburg, Germany) to avoid toothbrush abrasion or any alterations during performance of the de-/remineralisation cycling. After the experimental procedure (20 days), surface profiles were recorded again and enamel wear was calculated using a custom made software able to perform superimposition of the baseline profiles and follow-up profiles. Superimposition of the two profiles was achieved by overlaying the reference areas (area outside the two reference lines). The step height between the baseline profile and follow-up profile in the area of the treated surface was considered as enamel wear. In case the assessed wear per profile was below the measurement limit of the profilometer (0.105 μm) [27], the value for this profile was set to 0.000 μm. Enamel wear of each specimen was calculated by averaging the values of the five respective profiles and the mean loss in each group was gathered by averaging the values all specimens of the associated group.

### Statistical analysis

The dataset was statistically analysed using one-way analysis of variance (ANOVA) and residuals were checked for normality and variance homogeneity. After post-hoc pairwise comparison (G1–5), *p*-values were corrected according to Tukey`s HSD (honest significant difference). The level of statistical significance was set at 5%. All statistical analyses and plots were computed with the statistical software R [28].

## Results

**Fig. 2:**
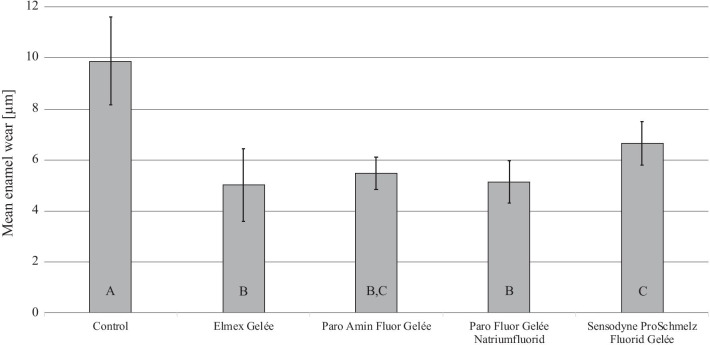
Enamel wear (μm) (mean ± SD) after 20 days of de-/remineralisation cycling for the control (G1) and different fluoride gels [Elmex Gelée (G2); Paro Amin Fluor Gelée (G3); Paro Fluor Gelée Natriumfluorid (G4); Sensodyne ProSchmelz Fluorid Gelée (G5)]. Significantly different values (*p* < 0.05) are marked with different capital letters

The mean enamel loss of each group (G1–5) after 20 days of de-/remineralisation cycling is illustrated in Fig. 2. All five test groups showed more or less severe enamel wear. The overall highest mean wear of enamel (9.88 ± 1.73 µm) was observed in the control group (G1) and was significantly higher (*p* < 0.001) than in all other groups where fluoride gels were applied. Elmex Gelée (G2; 5.03 ± 1.43 µm), Paro Amin Fluor Gelée (G3; 5.47 ± 0.63 µm, *p* = 0.918) and Paro Fluor Gelée Natriumfluorid (G4; 5.14 ± 0.82 µm, *p* > 0.999) showed the best protection from hydrochloric acid induced erosion. Although enamel wear in G5 (Sensodyne ProSchmelz Fluorid Gelée; 6.64 ± 0.86 µm) was significantly lower than in the control group (*p* < 0.001), it still was significantly higher compared to G2 (*p* = 0.028) and G4 (*p* = 0.047).

## Discussion

Enamel specimens in this in-vitro-study were prepared from bovine incisors, which have been used and discussed in multiple studies investigating erosive/abrasive wear of dental hard tissues and were shown to be a suitable substitute for human enamel [26, 29, 30]. Surface wear was measured using profilometric surface analysis, which is proven to be a reliable and accurate method in order to quantify erosive/abrasive enamel wear and has been used and discussed in numerous other studies investigating this issue [14, 31]. The reflux simulating cycling model and all applied parameters aimed to simulate realistic clinical conditions as they might occur in a reflux patient. Therefore, a HCl perfusion was used to imitate regurgitation of gastric fluid reaching the oral cavity. The quantity (11 times a day during 12 h) and duration (30 s) of the acid attacks, as well as flow rate of 2 ml/min and pH-value 3.0 [32] also tried to reflect realistic conditions although these parameters are known to vary from patient to patient thus limiting standardized comparison [24, 25].

Based on recommendations for patients with signs of dental erosion, daily toothbrushing was performed before erosion and one hour after the last erosive attack to minimize wear of freshly eroded, softened enamel with a higher susceptibility for brushing abrasion [33]. The quantity (2 times/day), as well as the amount (n = 15), frequency (1 stroke/second) and application force (2.0 N) of the performed brushing strokes were based on recommendations by Wiegand and Attin [34]. The required overall duration of the applied cycling model (20 days) to get clear, measurable results was verified in a study with similar set up [14].

However, it has to be considered that the occurrence of gastroesophageal reflux in-vivo is not limited to a defined number and time of a day and may also happen while sleeping at night [35]. As no reflux was simulated during 12 h (overnight) in this study, this might be a limitation of the present cycling model. Furthermore, gastric fluid is mainly, but not only composed of HCl and may contain various enzymes such as the proteolytic enzyme pepsin [36]. Other than in a previous study [37], the acid was not enriched with pepsin in this study, as the amount of organic matrix in enamel is much lower compared to dentine and no influence of pepsin admixture on the erosive/abrasive tooth wear was observed even for dentin [38]. Other modifying factors such as pellicle formation, bacteria and fluoride in saliva and plaque fluid were also not regarded in this study. Additionally, it has to be considered that the storage of specimens in artificial saliva between the brushing periods does not adequately imitate *in-vivo-*mineralising processes [39].

In general, saliva might enhance the abrasive wear resistance and support remineralisation through calcium und phosphate precipitation and thus lead to a stabilisation of eroded enamel [40]. However, saliva induced remineralisation must be regarded as a slow process with mineral gain mainly taking place in the surface layer of the lesion [41]. This might be the reason, why only a minor remineralising effect of previously eroded enamel [42] and still increased susceptibility to abrasion of previously eroded enamel after a remineralisation period of one hour [43] is described in literature. Another study reports partial re-hardening of softened enamel surface within two hours of salivary exposure but no significant further remineralisation after 12 h [44]. Thus, it is questionable if the specimen storage in artificial saliva between the erosive attacks and during 12 h (overnight) in this study might have led to a notable remineralisation.

After 20 days of daily application, all investigated amine/sodium and sodium fluoride gels (12.500 ppm) were able to significantly reduce gastroesophageal reflux induced loss of enamel. This finding is in agreement with the results of other studies investigating the potential of different fluoride gels to protect from DE [45, 46]. Basically, the protective effect of fluoride can be attributed to the formation of a calcium-fluoride (CaF_2)_ layer on the enamel surface enabling a resistant and protective surface coating against the attacking acid. The layer functions as a mechanical barrier and at the same time provides a reservoir of minerals able to buffer or deplete hydrogen ions from the acid [22]. During an acidic attack, fluoride is released from the CaF_2_ layer and can be incorporated into tooth mineral by forming fluorapatite or fluorohydroxyapatite with decreased susceptibility to further dissolution [22].

The amine/sodium fluoride gel in G2, the amine/sodium fluoride gel in G3 and sodium fluoride gel in G4 showed the overall best protection in this study. Enamel wear in G5 (sodium fluoride gel, Sensodyne ProSchmelz Fluoride Gelée) was significantly higher compared to the amine/sodium fluoride gel in G2 and the sodium fluoride gel in G4. The reasons for these differences remain unclear. It is reported that, at the same concentrations, amine fluoride is likely to be more effective than sodium fluoride to protect enamel from acid [22]. Nevertheless, the same kind of fluoride (sodium fluoride) was applied in G4 which showed significantly less enamel wear. Therefore, the differences might rather not or not only be attributed to the compound of fluoride. Anyway, the potential to protect from gastroesophageal reflux induced DE in G5 still was significantly higher compared to the untreated control group and is in conformance with other studies describing a protective effect of sodium fluoride [47, 48].

The concentration of fluoride and pH-value may also have an influence. It is evident, that a low pH and high fluoride concentration support the uptake of fluoride into dental hard tissues and the formation of a CaF_2_ layer [49]. All tested fluoride gels in this study had the same high fluoride concentration of 12.500 ppm which has been proven to be effective in reducing erosive tooth wear [45]. Regarding the differing pH-values of the gels, it has to be taken into consideration that enamel specimens in this study were eroded before the fluoride gels were applied. Erosion causes an enlargement of the enamel surface which enables higher fluoride uptake [50], thus making the pH of the applied fluorides less important.

Besides fluoride, chitosan and stannous chloride have recently been added to several commercial anti-erosive toothpastes and mouthwashes as they were discovered to be potent reactants with hydroxyapatite and may further reduce the solubility of dental hard tissue thus enabling additional anti-erosive properties [51]. However, these active ingredients were not contained in any of the investigated products so that the significant reduction of enamel softening observed in this study might without a doubt be attributed to the fluoride compounds. The effects of fluoride on the oral cavity and the entire human organism are well known and investigated [52]. Therefore, the use of a 12.500 ppm fluoride gel once a day can be recommended in order to reduce GERD-induced dental erosion.

## Conclusions

Within the limitations of this in-vitro-study, it might be concluded that erosive/abrasive tooth wear, caused by frequent HCl exposure in the course of gastroesophageal reflux and toothbrushing, can significantly be reduced through daily application of 12.500 ppm fluoride gels, irrespective of fluoride compound or pH-value.

## Data Availability

The datasets used and/or analysed during the current study are available from the corresponding author on reasonable request.

## Abbreviations

DE: Dental erosion
GERD: Gastroesophageal reflux disease
